# Anaesthetic Considerations for Twin Delivery in a Patient With Osteogenesis Imperfecta Type III: A Case Report

**DOI:** 10.7759/cureus.118667

**Published:** 2026-10-27

**Authors:** Sneha E Eapen, Bronte Bang Ting Jong, Leonard Wei Wen Loh

**Affiliations:** 1 Department of Anesthesiology, Singapore General Hospital, Singapore, SGP; 2 Department of Anaesthesia, Ministry of Health, Singapore, SGP

**Keywords:** brittle bone disease, connective tissue disease, obstetric anaesthesia, osteogenesis imperfecta, twin pregnancy

## Abstract

Osteogenesis imperfecta is an uncommon, inherited connective tissue disease, which is rare in pregnancy and presents multiple challenges to perinatal and anaesthetic care. Caesarean section in these patients is complex, often performed preterm and demands careful coordination between multiple disciplines. A 27-year-old woman at 26 weeks’ gestation of a spontaneous twin pregnancy presented for elective caesarean section for worsening respiratory failure. General anaesthesia with total intravenous anaesthesia was used in view of anticipated prolonged surgery and difficult regional anaesthesia. The delivery was largely uneventful, and the patient recovered without major complications. The twins experienced multiple complications of prematurity. This case highlights the importance of multidisciplinary planning in achieving favourable maternal and foetal outcomes in high-risk pregnancies.

## Introduction

Osteogenesis imperfecta (OI) is an inherited connective tissue disease with several described types [1]. Uncommon in pregnancy, it presents several challenges to perinatal care due to multisystem involvement. Multiple pregnancy further complicates the situation, increasing risks to mother and foetuses.

There are several considerations in anaesthetising a patient with OI for caesarean section (CS).

## Case presentation

The patient is a 27-year-old women of Malay ethnicity with a spontaneous monochorionic diamniotic twin pregnancy. Her medical history includes OI Type III, complicated by recurrent fractures, kyphoscoliosis (as demonstrated in Figure 1), restrictive lung defect, left-sided hearing loss, dentinogenesis imperfecta, and subclinical hyperthyroidism. She reported five previous fractures over both upper and lower limbs, all of which were managed conservatively. Premorbidly, the patient was wheelchair-bound with very low effort tolerance. She could perform some basic activities of daily living independently but needed assistance otherwise.

**Figure 1:**
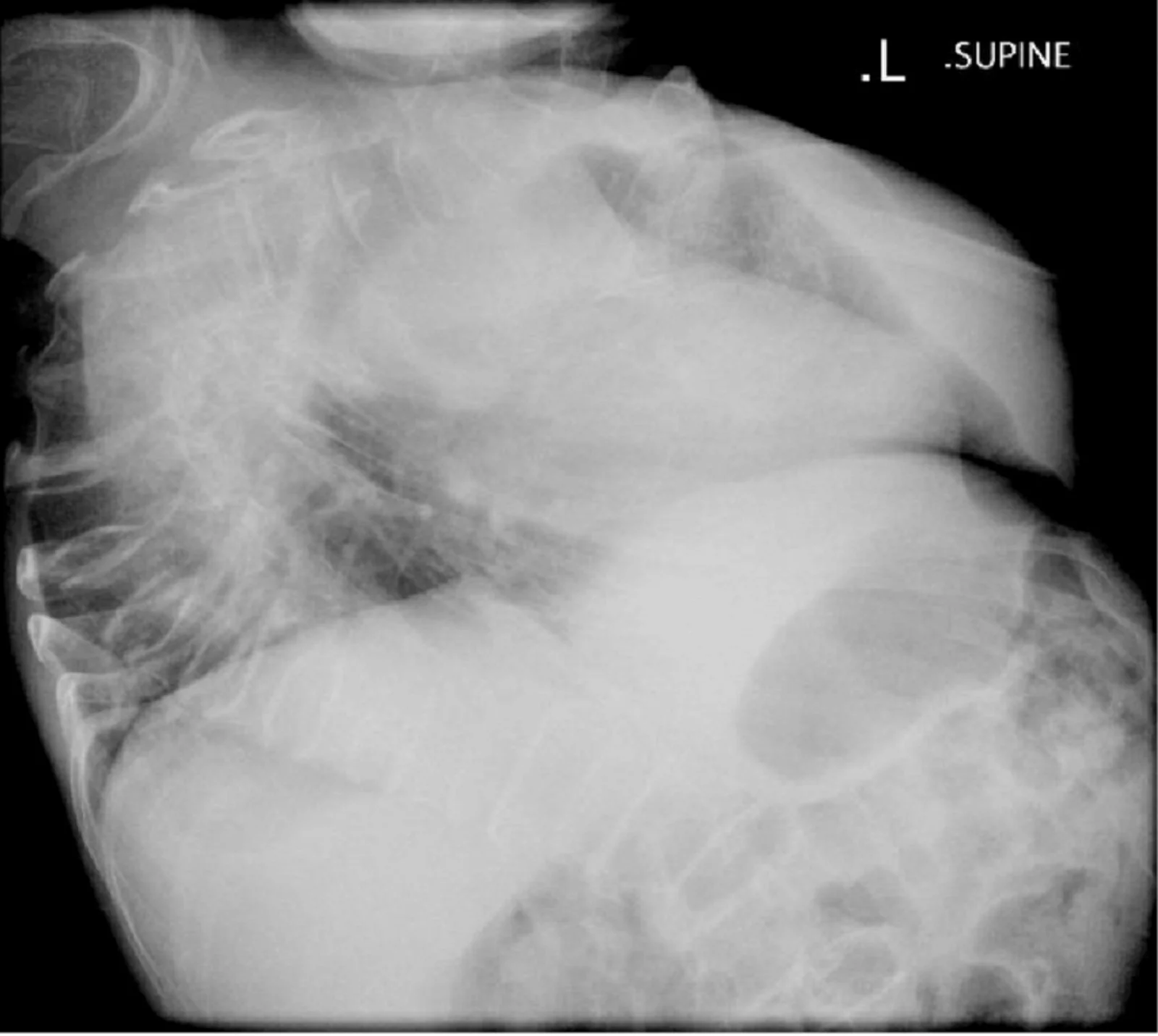
Prenatal supine posterior-anterior chest X-ray of the patient

She first presented to a maternity hospital at eight weeks’ gestational age and was referred to our tertiary centre at 14 weeks’ gestation. A high-risk obstetric anaesthetic consultation at 16 weeks’ gestation documented her weight as 38 kg, height as 87 cm, and torso length as 47 cm.

During her first trimester, she became progressively dyspnoeic. She could not lie flat at 21 weeks’ gestation and was visibly tachypnoeic even upright by 23 weeks, with room-air SpO_2_ 93%. She was progressively tachycardic with a heart rate of 120-130 beats/min from a baseline <100 beats/min but declined admission. Spirometry revealed a severe restrictive lung disease pattern with a forced expiratory volume in 1 second of 0.4 L (37% predicted) and forced vital capacity of 0.4 L (35% predicted). At 23 weeks’ gestation, she started nocturnal non-invasive ventilation and daytime O_2_ 2 L/min, aiming for SpO_2_ >95%. The patient and her husband were counselled about the high-risk nature of the pregnancy and the risks to herself and the foetuses but refused termination. Antenatal genetic foetal testing was negative for OI.

Multiple multidisciplinary team meetings involving obstetricians, anaesthetists, neonatologists, respiratory physicians, and intensivists took place to discuss her care.

At 25 weeks’ gestation, she was admitted from the obstetric clinic for worsening breathlessness. It was decided to perform an elective CS at 26 weeks after completing dexamethasone therapy for foetal lung maturation.

On the day of CS, her weight was 18.9 kg, significantly lower than recorded in early pregnancy. As the patient’s nutritional and fluid status appeared appropriate and largely unchanged from previous visits, we suspect that there may have been a measurement or recording error previously. Her weight at several post-delivery visits was recorded between 18 and 19 kg.

In the induction room, 18-gauge intravenous cannulae were set on both hands, and a 20-gauge intra-arterial cannula was sited in her left radial artery. A paediatric blood pressure cuff was placed on her left arm, and bispectral index (BIS) monitoring was applied. 

In view of a challenging central neuraxial block and potentially prolonged surgery due to twin pregnancy, we opted for general anaesthesia (GA).

Preoperative airway examination revealed a Mallampati score [2] of 1, adequate mouth opening, and thyromental distance with full cervical range of motion. However, given her small size, fracture risks, and possibly altered airway anatomy, there were concerns about securing her airway once anaesthetised. After topicalisation with oral 10% lignocaine 0.3 mL and nasal co-phenylcaine 0.1 mL, an awake examination using a McGrath video laryngoscope (MEDTRONIC, USA) revealed a grade 1 laryngeal view.

Anaesthesia was induced using propofol target-controlled infusion (Schneider effect site model) and 1 mg/kg rocuronium to facilitate intubation with a size 6.5 Portex endotracheal tube. Maintenance used total intravenous anaesthesia (TIVA) guided by BIS monitoring. She was positioned supine with careful padding at all bony prominences. We ventilated her in pressure-control mode with an inspiratory pressure (Pi) of 18 cmH_2_O, a respiratory rate of 12 breaths/min, and a positive end-expiratory pressure (PEEP) of 0, achieving tidal volumes (TVs) of 200-220 mL and a peak pressure (Ppeak) of 19 cmH_2_O.

A classical CS approach with a lower midline laparotomy was necessary due to lower segment access limitations from the patient’s very small stature and skeletal deformities. After delivery of both foetuses, uterine atony caused postpartum haemorrhage (PPH) of 800 mL. She required transfusion of 276 mL of red blood cells (RBCs) and received 50 mcg of carbetocin with an additional 10 mcg bolus shortly after. B-Lynch sutures were placed. However, suboptimal uterine tone persisted, and oxytocin 3 IU/hour was continued postoperatively.

Perioperative analgesia included 50 mcg IV fentanyl, 300 mg IV paracetamol, and bilateral rectus sheath catheters infusing 0.2% ropivacaine at 4 mL/hour. Patient-controlled analgesia (PCA) with fentanyl was given postoperatively.

The surgery lasted 1 hour 53 minutes. In view of her restrictive lung disease and PPH, she was transferred to the intensive care unit (ICU) postoperatively. She received 2 units of RBCs, 1 unit of platelets, 250 mL of fresh frozen plasma, and 10 units of cryoprecipitate.

In the ICU, she was ventilated with assist-control pressure ventilation with a Pi of 13 cmH_2_O, a PEEP of 0, generating a TV of 230-250 mL. On post-operative day (POD) 1, she was extubated to bi-level positive airway pressure (BiPAP) ventilation. She was weaned to nasal prongs O_2_ and transferred to the general ward on POD4. PCA fentanyl was discontinued on POD4, and the patient returned home on POD12. Both babies required the neonatal ICU, having suffered multiple complications of prematurity.

## Discussion

OI is a congenital connective tissue disorder affecting the synthesis of Type I collagen. It often results from mutations in the *COL1A1* and *COL1A2* genes [3], which code for Type I collagen’s alpha-1 and alpha-2 chains. Inheritance may be autosomal dominant or recessive, depending on the gene mutation. Sillence originally classified four types of OI based on clinical features and gene mutations [4]. Subsequent nomenclature revisions, with the discovery of new genetic defects, have expanded the classification to include Types V-XIV [5].

Phenotypically, Type II and III are more severe; Type II OI is often lethal in utero or shortly after birth due to severe fractures and respiratory failure. Type III is associated with early, numerous fractures leading to progressively worsening deformities, including significant kyphoscoliosis, short stature, and thoracic deformities [6]. Patients with Type I OI have the mildest form with a variable fracture rate, while Type IV OI causes moderate bone fragility and moderate deformities.

Among parturients with OI, Type IV is most frequent, with largely singleton pregnancies. Existing literature on parturients with OI consists mostly of case reports on singleton pregnancies and in patients with less severe phenotypes of OI [7,8].

The multisystem effects of OI raise several concerns when providing anaesthesia, as detailed in Table 1. These were compounded in our patient by a twin pregnancy with an increased likelihood of prolonged surgery, uterine atony, and consequent PPH.

**Table 1:** Considerations in anaesthetising a patient with osteogenesis imperfecta for caesarean section

Category	Organ system	Considerations
Anaesthetic	Airway	Possible difficult airway -- difficult bag-mask ventilation and intubation due to limited cervical spine and mandibular range of motion. Increased risk for odonto‐axial dislocation. Brittle teeth (dentinogenesis imperfecta). Weak cervical and mandibular bones
	Respiratory	Respiratory failure from deformed thorax and kyphoscoliosis, resulting in restrictive lung deficit
	Cardiovascular	Valvular abnormalities (mitral regurgitation and aortic regurgitation). Atrial septal defects. Ventricular septal defects. Aortic root dilatation
	Metabolic	Risk of malignant hyperthermia-like response. Difficulty with temperature regulation due to altered body surface area and prone to developing hyperthermia
	Musculoskeletal	Kyphoscoliosis and spondylolisthesis -- difficult regional anaesthesia. Vertebral deformities. Risk of fractures from repeated blood pressure cuffing. Risk of fractures from succinylcholine-induced fasciculations. Risk of fractures from low-energy incidents, e.g., leaning, instruments, or during transfer
	Haematological	Associated with low platelet counts and platelet dysfunction. Reduced Factor VII activity. Increased capillary fragility. Impaired platelet aggregation
Obstetric		Increased risk of antepartum haemorrhage, including placental abruption. Increased risk of preterm delivery. Increased risk of intrauterine growth restriction (IUGR). Increased incidence of abnormal presentation. Cephalopelvic disproportion. Risk of uterine rupture and pelvic fractures. Higher incidence of intrapartum and postpartum haemorrhage (PPH)
Surgical		Difficult access to the uterus via lower segment (potentially requiring classical CS). Challenging positioning due to long bone deformities. Difficult haemostasis due to limited access

While sole regional anaesthesia has been described for patients with severe OI undergoing CS, this is limited to singleton deliveries [9,10]. Preoperative ultrasound imaging of our patient’s lumbar spine revealed severe rotation, with difficult identification of the midline. We worried a block might be patchy or unilateral given the degree of distortion. Impending respiratory failure and potentially prolonged surgery also favoured a general anaesthetic technique. TIVA could help avoid non-malignant hyperpyrexia [11].

Given the fracture risk and potentially difficult bag-mask ventilation and intubation, awake video laryngoscopy helped assess airway anatomy and determine whether awake intubation was necessary. Confirmation of a good laryngeal grade allowed us to proceed with asleep intubation after rapid sequence induction, allaying the patient’s extreme anxiety.

Respiratory physicians came into OT to observe the case, with particular attention to intraoperative ventilatory mode selection and parameter titration. A lung-protective ventilation strategy was employed, targeting TV of 6 mL/kg of ideal body weight (IBW). However, IBW estimation was likely imprecise given the patient’s short stature. Pressure control ventilation with a Ppeak of 19 cmH₂O provided sufficient gas exchange while minimising the risk of ventilator-induced lung injury. Fluctuations in intrathoracic pressure during the surgery intermittently affected lung volumes and compliance.

The patient transitioned to BiPAP on POD1 to facilitate ventilator weaning. This patient’s poor intrinsic chest wall compliance, with low TV and increased elastic work of breathing, predisposed her to rapid shallow breathing and respiratory muscle fatigue. These were exacerbated postoperatively by residual anaesthetic effects, diaphragmatic dysfunction, supine positioning, abdominal splinting, and pain-related inhibition of deep inspiration and coughing, further reducing her functional residual capacity (FRC) and promoting atelectasis. BiPAP is particularly beneficial in restrictive pathophysiology, as it augments spontaneous TV, maintains FRC, and recruits atelectatic lung segments [12]. Early extubation to non-invasive ventilation also reduces weaning failure and reintubation in high-risk patients, shortens duration of mechanical ventilation, and decreases ICU length of stay compared with conventional invasive weaning strategies [13,14].

Circulation management was a challenge in this patient, given her markedly reduced circulating volume. While primary PPH following CS is defined as a blood loss of >1,000 mL, our patient’s estimated blood volume was 1.4 L, of which she lost >50%. Additionally, there was a possibility of a bleeding diathesis, as the patient reported easy bruising. We also needed to be cognizant of the potential for fluid overload given her small size.

As there is no data on carbetocin dosing in adults <30 kg [15], we reduced the initial dose and repeated 10 mcg aliquots until adequate uterine tone was observed.

It was essential to balance effective analgesia with the risk of opioid-induced respiratory depression in a patient with pre-existing respiratory dysfunction. Heightened pain was anticipated given the lower midline laparotomy incision. While PCA morphine is prescribed as 0.1 mg/kg in paediatric settings, such individualised dosing was not familiar to the adult ICU. We opted for PCA fentanyl instead, with 20 µg boluses. To further reduce opioid requirements and facilitate early mobilisation, a multimodal analgesic regimen with regular paracetamol and rectus sheath catheters proved useful.

## Conclusions

Multidisciplinary team discussion and advanced planning were paramount in the safe and holistic perinatal care of this patient with rare multisystem disease and multiple pregnancy. Although the choice of GA did not allow the patient to interact with her babies immediately after delivery, the chosen technique allayed the patient’s significant anxiety regarding her surgery while enabling the team to focus on respiratory and haemodynamic management, guided by point-of-care testing, to optimise maternal and foetal outcomes.
